# Risk factors for and outcomes after unintended pregnancy: Evaluation in a contemporary cohort

**DOI:** 10.1002/pmf2.70075

**Published:** 2025-07-11

**Authors:** Nigel Madden, Timothy Wen, Madeline Perry, Ashish Premkumar, Lynn M. Yee

**Affiliations:** ^1^ Division of Maternal‐Fetal Medicine Northwestern University Feinberg School of Medicine Chicago Illinois USA; ^2^ Beth Israel Deaconess Medical Center Division of Maternal‐Fetal Medicine Boston MA USA; ^3^ Division of Biomedical Informatics and Division of Maternal‐Fetal Medicine University of California San Diego School of Medicine La Jolla California USA; ^4^ Department of Obstetrics and Gynecology University of Pennsylvania Perelman School of Medicine Philadelphia PA USA; ^5^ Division of Maternal‐Fetal Medicine University of Chicago School of Medicine Chicago Illinois USA

**Keywords:** health policy, maternal morbidity, pregnancy intention, public health, unintended pregnancy

## Abstract

**Introduction:**

Prior research has demonstrated associations between unintended pregnancy and adverse birth outcomes. The recent restrictions on abortion access may result in a rise in unintended pregnancy. Our objective was to comprehensively evaluate risk factors for and outcomes after unintended pregnancy in a contemporary cohort.

**Methods:**

Cross‐sectional analysis of the Pregnancy Risk Assessment Monitoring System (2016–2020) respondents with singleton live births. The primary exposure was unintended pregnancy, including respondents who reported being unsure, had mistimed, or had undesired pregnancies. Models evaluated the association between unintended pregnancy and social determinants of health, selected using the US Department of Health and Human Services Healthy People 2030 conceptual framework. Multivariable regression models estimated the independent association between pregnancy intention and outcomes, including maternal healthcare utilization and maternal and neonatal outcomes. Secondary analyses evaluated the three subgroups of unintended pregnancies.

**Results:**

In this weighted population of 8.9 million respondents, 40.0% reported unintended pregnancy. Social determinants of health associated with unintended pregnancy included young age, non‐Hispanic Black race, Hispanic ethnicity, being unmarried, more prior live births, more income dependents, and lower household income. Unintended pregnancy was associated with decreased healthcare utilization, including lower odds of prenatal care in the first trimester (aOR 0.60, 95% CI 0.57–0.63), adequate prenatal care (aOR 0.75, 95% CI 0.72–0.78), and postpartum visit attendance (aOR 0.88, 95% CI 0.83–0.94), but higher odds of using postpartum contraception (aOR 1.44, 95% CI 1.38–1.50). Unintended pregnancy was also associated with higher odds of adverse maternal outcomes including perinatal depression (during pregnancy, aOR 1.53, 95% CI 1.43–1.65; postpartum, aOR 1.29, 95% CI 1.22–1.37), and intimate partner violence (aOR 1.44, 95% CI 1.26–1.65), as well as lower odds of breastfeeding more than 3 months (aOR 0.90, 95% CI 0.86–0.94), but was not associated with neonatal outcomes. In secondary analyses, the three unintended pregnancy subgroups (mistimed, unsure, and undesired) were each individually associated with higher odds of adverse outcomes compared to intended pregnancy. Undesired pregnancy had the strongest associations with adverse outcomes, including higher odds of hypertensive disorders of pregnancy (aOR 1.26, 95% CI 1.14–1.40).

**Conclusion:**

Unintended pregnancy is associated with adverse maternal outcomes in this contemporary cohort. Findings highlight populations that are vulnerable to the adverse consequences of unintended pregnancy, which is of particular importance in the era of restricted reproductive freedom.

## 1 INTRODUCTION

In 2019, 41.6% of pregnancies in the United States (US) were unintended [1]. Although this percentage has been declining, it is still far above the goal set by the Department of Health and Human Services (DHHS) Healthy People 2030 campaign [2]. Previous research, including a recent systematic review and meta‐analysis, has demonstrated an association between unintended pregnancy and adverse maternal and neonatal outcomes [3, 4, 5, 6]. As it is well established that minoritized and disadvantaged populations are more likely to have unintended pregnancies [7], individuals from these groups are at increased risk of adverse sequelae of such pregnancy circumstances.

The restrictions on abortion access because of the US Supreme Court decision in *Dobbs v. Jackson Women's Health Organization*, which overturned the constitutional protection afforded by *Roe v. Wade*, may result in an increase in continuation of unintended pregnancy, particularly among socially disadvantaged populations who may be less able to overcome the economic and logistical barriers of abortion care in ban states [8, 9, 10, 11]. This changing landscape of reproductive autonomy necessitates a critical understanding of the factors that increase the risk for unintended pregnancy and the potential complications when these pregnancies are continued. Existing population‐level research uses older data or analyzes limited outcomes [3, 4, 5, 6]. As such, our objectives were (1) to evaluate social determinants of unintended pregnancy and (2) to assess the association between unintended pregnancy and a comprehensive set of maternal and neonatal outcomes (maternal healthcare utilization, maternal health outcomes, and neonatal outcomes) in a cross‐sectional, contemporary cohort of individuals with a live birth.

## 2 MATERIALS AND METHODS

This study uses population‐weighted data from Phase 8 (2016 to 2020) of the Pregnancy Risk Assessment Monitoring System (PRAMS) from the Centers for Disease Control and Prevention. PRAMS is a population‐based surveillance system administered to individuals who recently had a live birth. Pregnancies that ended in miscarriage, abortion, or stillbirth are not represented. The questionnaires are completed by mail or telephone interview and collect data on maternal health behaviors, attitudes, and experiences before, during, and shortly after pregnancy [12]. Survey responses are linked to birth certificate information. This study sample included individuals with singleton live births from 47 states, Washington, DC, and Puerto Rico. California, Idaho, and Ohio were not included as they did not report to PRAMS during the study period. South Carolina did report to PRAMS during the study period, however their data is only available by direct request from the site and cannot be merged with the PRAMS data file, as such this state was also excluded from our analysis. In the year 2016 alone an additional nine states and territories did not report to PRAMS (Arizona, Indiana, Kansas, Kentucky, Montana, Nevada, North Dakota, Washington, DC, and Puerto Rico). Additionally, PRAMS sets a minimum overall response rate threshold for the release of data for each year and the data from states that do not meet this threshold in any given year is not available within the dataset. For the years 2016–2017, the response rate threshold for release was 55% and for the years 2018–2020 it was 50%. Table S1 reviews which of the included states met the response rate threshold for each of the years of this study. We additionally excluded respondents with missing pregnancy intention data. Sociodemographic and outcomes variables were evaluated for missingness and four variables (number of income dependents, pre‐pregnancy body mass index [BMI], adequacy of prenatal care, and breastfeeding duration) were found to have > 3.0% missing data. Individuals who were missing one or more of these variables were also excluded from the analysis (total of 12.5% of observations excluded). PRAMS is deidentified and publicly available data, therefore the study was exempt from IRB approval.

Pregnancy intention was assessed by the question “Thinking back to just before you got pregnant with your new baby, how did you feel about becoming pregnant?” Individuals who responded “I wanted to be pregnant sooner” or “I wanted to be pregnant then” were categorized as having an intended pregnancy. Consistent with prior literature [3, 4, 13], all other responses were classified in the “unintended” pregnancy group. The unintended pregnancy group included subgroups of participants who reported pregnancies that were undesired, mistimed, or who were unsure about the pregnancy. Individuals who responded, “I didn't want to be pregnant then or at any time in the future” were categorized has having an undesired pregnancy. Individuals who responded, “I wanted to be pregnant later” were categorized as having a mistimed pregnancy. Individuals who responded, “I wasn't sure what I wanted” were categorized as being unsure about the pregnancy.

For our first objective, we selected potential determinants of pregnancy intention based on the US DHHS Healthy People 2030 domains of social determinants of health. Variables were derived from the PRAMS respondent questionnaire and linked birth certificate data. We evaluated factors in four domains: education access and quality (years of education defined as less than 12 years, 12 years, or more than 12 years), social and community context (marital status, number of prior live births), economic stability (household income per year, number of income dependents), and healthcare access and quality (pre‐pregnancy insurance provider, pre‐pregnancy BMI, smoking history, and history of pre‐gestational diabetes, chronic hypertension, or depression). Given a significant amount of missing data (5.2%), household income level was regression‐imputed for respondents with missing data using respondent age, race and ethnicity, marital status, and education. Additional maternal sociodemographic factors (age, race and ethnicity) were evaluated given prior research demonstrating associations with pregnancy intention [5, 7] likely, in part, due to their associations with other social determinants of health such as exposure to discrimination and racism. Maternal racial and ethnic identities are as reported on the neonatal birth certificates and were categories as Asian, Hispanic, non‐Hispanic Black, non‐Hispanic White, and a “none of the above” category which contained smaller racial and ethnic groups including individuals who identified as American Indian, Hawaiian, Alaska native, or mixed race.

For the second objective, the primary exposure was intended versus unintended pregnancy. Outcomes of interest included those assessing maternal healthcare utilization and maternal and neonatal outcomes. For evaluation of healthcare utilization, the question “What type of healthcare visit did you have in the 12 months before you got pregnant with your new baby?” with the selection “Regular checkup at my OB/GYN's office” was used to assess attendance of a pre‐pregnancy obstetrician/gynecologist (OB/GYN) visit. The question “During any of your healthcare visits in the 12 months before you got pregnant, did a doctor, nurse, or other healthcare worker do any of the following things?” with the selections “Talk to me about my desire to have or not have children” and “Talk to me about how I could improve my health before a pregnancy” were used to assess pre‐pregnancy discussions with healthcare clinicians. Adequacy of prenatal care was assessed using the Kessner Index which categorizes prenatal care as adequate, intermediate, or inadequate based on the month of pregnancy in which prenatal care began, the number of prenatal visits, and the length of pregnancy [14]. Other healthcare utilization outcomes included whether prenatal care was initiated in the first trimester, receipt of the influenza vaccine, postpartum visit attendance, postpartum contraception use, and use of long‐acting reversible contraception (LARC).

Maternal health outcomes included receiving a diagnosis of a hypertensive disorder of pregnancy including either gestational hypertension or preeclampsia, depression diagnosed during pregnancy, intimate partner violence (IPV) that occurred during pregnancy, depression diagnosed postpartum, and breastfeeding duration of greater than three months. Neonatal outcomes including preterm birth less than 37 weeks’ gestation, preterm birth less than 34 weeks’ gestation, small for gestational age (birth weight less than 10th percentile), low birth weight (birth weight less than 2500 g), and neonatal length of stay for greater than 5 days.

For both the first and second objectives, the primary analysis was completed comparing the intended pregnancy group to the unintended pregnancy group which included individuals in the three unintended pregnancy subgroups (mistimed, unsure, and undesired). This division of groups is consistent with prior literature. However, it is understood that desiring a pregnancy later or being ambivalent about pregnancy may be different from having an undesired pregnancy, and as such, these subgroups may be associated with different outcomes. In order to explore these differences further, secondary analyses were completed for both of the objectives evaluating our results in the three subgroups within the “unintended” pregnancy category.

### 2.1 Statistical analysis

For our first objective, we first descriptively compared social determinants of pregnancy intention in individuals with intended versus unintended pregnancy using chi‐square tests. These social determinants were then included in a comprehensive multivariable logistic regression model to evaluate the independent association of each risk factor with unintended pregnancy. A second model examined these relationships in the subgroup of respondents with undesired pregnancy, compared to those with intended pregnancy.

For our second objective, maternal healthcare utilization outcomes, maternal health outcomes, and neonatal outcomes were first compared between those with intended versus unintended pregnancies using chi‐square tests. Unadjusted and adjusted logistic regression models were fit to assess the association between the primary exposure of pregnancy intention, with intended pregnancy as the referent group, and these outcomes, adjusting for potential confounders including maternal age, race and ethnicity, education, marital status, number of prior live births, annual household income, number of income dependents, pre‐pregnancy insurance, maternal body mass index, smoking, pregestational diabetes, chronic hypertension, and history of depression. A secondary analysis (shown in the Supporting Information) repeated these bivariable and multivariable analyses in the three unintended pregnancy subgroups using intended pregnancy as the referent group.

The PRAMS survey uses a complex sampling design to estimate representative state populations based on birth certificate data and the survey weighting methods reflect specific population strata and nonresponse rates. All results are reported as population‐weighted estimates using the survey module of Stata Version 18 (College Station, TX).

## 3 RESULTS

There were 172,565 respondents who met inclusion criteria, representing a weighted population of 8.9 million individuals, with a live birth from 2016 to 2020. Of these, 60.0% reported the pregnancy had been intended and 40.0% unintended, of which 6.2% were undesired, 18.7% were mistimed, and 15.1% reported they were unsure.

For our first objective, all determinants demonstrated significant differences across pregnancy intention groups, except for pre‐gestational diabetes (Table 1). Multivariable modeling of these social determinants of pregnancy intention demonstrated many persistent findings (Table 2). Maternal sociodemographic factors associated with unintended pregnancy included age less than 20 years (aOR 2.73, 95% CI 2.45–3.05), non‐Hispanic Black race (aOR 1.87, 95% CI 1.77–1.97), and Hispanic ethnicity (aOR 1.17, 95% CI 1.11–1.24). Social and economic factors associated with unintended pregnancy included being unmarried, having more prior live births, having lower income, and having a higher number of income dependents (Table 2). The measures of healthcare access and quality that were associated with unintended pregnancy included Medicaid health insurance (aOR 1.18, 95% CI 1.12–1.25) or no insurance (aOR 1.32, 95% CI 1.24–1.58), smoking history (aOR 1.51, 95% CI 1.43–1.58), and a history of depression (aOR 1.39, 95% CI 1.32–1.47). A subgroup analysis evaluating factors associated with *undesired* pregnancy showed similar results (Table 2), with the addition that having 2 or more (aOR 4.05, 95% CI 3.54–4.63) or 3 or more (aOR 5.40, 95% CI 4.64–6.27) prior live births were both strongly associated with undesired pregnancy, as well as age > 40 years, obesity, and chronic hypertension.

**TABLE 1:** Social determinants of pregnancy intention in individuals with a live birth.^a^, ^b^

Social determinant categories and factors	Intended (60.0%)	Unintended (40.0%)
*Maternal sociodemographic*		
Age (years)		
< 20	1.5	7.2
20–30	42.3	54.3
30–40	52.5	35.7
≥ 40	3.6	2.8
Race and ethnicity		
Asian	6.0	4.1
Hispanic	14.2	18.2
Non‐Hispanic Black	9.2	22.7
Non‐Hispanic White	67.1	50.0
None of the above^c^	3.5	4.7
*Education access and quality*		
Education		
Less than 12 years	7.2	13.2
12 years	18.7	32.0
More than 12 years	74.1	54.8
*Social and community context*		
Unmarried	23.4	57.1
Parity		
Nulliparity	41.2	36.2
1 prior live birth	36.8	28.5
2 prior live births	14.0	19.5
3 or more prior live births	8.0	15.8
*Economic stability*		
Income category (per year)		
< $20,000	14.6	35.5
$20,000–40,000	16.4	24.5
$40,000–60,000	13.2	11.7
$60,000–85,000	13.1	7.7
> $85,000	42.8	20.1
Income dependents		
1 (self)	5.7	13.6
2	37.4	28.3
3	33.8	25.4
4	14.1	17.9
≥ 5	9.1	14.9
*Healthcare access and quality*		
Insurance (pre‐pregnancy)		
Private	61.2	32.4
Medicaid	17.0	35.6
Healthcare exchange	3.7	3.4
Other	8.7	13.3
None	9.3	15.3
Pre‐pregnancy BMI (kg/m^2^)		
< 18.5	2.9	3.8
18.5–25	47.3	41.4
25–29	26.0	25.8
≥ 30	23.9	29.1
Smoking history	11.4	23.3
Pre‐gestational diabetes	3.0	3.1
Chronic hypertension	4.6	5.9
Depression history	11.0	18.8

*Note*: Data presented as %.

Abbreviation: BMI, body mass index.

^a^ *N* = 172,565; weighted population 8,866,281 with live singleton births from 43 states and the District of Columbia, excluding Arizona, California, Idaho, Nevada, Ohio, South Carolina, and Texas.

^b^ All comparisons significant at *p* < 0.01, with the exception of pre‐gestational diabetes (*p* = 0.38).

^c^ The “none of the above” race and ethnicity category refers to individuals identified as American Indian, Hawaiian, Alaska native, or mixed race; these groups were collapsed due to insufficient sample size.

**TABLE 2:** Social determinants of unintended and undesired pregnancy in individuals with a live birth.^a^

Social determinant categories and factors	Unintended (40.0%)		Undesired (6.2%)	
	OR (95% CI)	aOR (95% CI)^b^	OR (95% CI)	aOR (95% CI)^b^
*Maternal sociodemographic*				
Age (years)				
< 20	3.62 (3.29–4.00)	**2.73 (2.45–3.05)**	1.18 (1.01–1.39)	**1.65 (1.37–1.99)**
20–30	1	1	1	1
30–40	0.53 (0.51–0.55)	**0.72 (0.69–0.75)**	1.10 (1.03–1.18)	**1.18 (1.09–1.27)**
≥ 40	0.61 (0.56–0.67)	**0.71 (0.64–0.78)**	2.45 (2.15–2.79)	**2.30 (1.98–2.68)**
Race and ethnicity				
Asian	0.90 (0.84–0.96)	1.37 (1.28–1.47)	0.93 (0.80–1.08)	1.43 (1.22–1.67)
Hispanic	1.71 (1.64–1.79)	**1.17 (1.11–1.24)**	1.45 (1.32–1.58)	**1.17 (1.06–1.30)**
Non‐Hispanic Black	3.29 (3.14–3.44)	**1.87 (1.77–1.97)**	2.65 (2.46–2.86)	**1.77 (1.62–1.95)**
Non‐Hispanic White	1	1	1	1
None of the above^c^	1.81 (1.69–1.93)	**1.21 (1.12–1.31)**	1.61 (1.41–1.84)	1.22 (1.06–1.41)
*Education access and quality*				
Education				
Less than 12 years	2.49 (2.36–2.62)	**0.77 (0.72–0.82)**	1.80 (1.63–1.98)	**0.72 (0.64–0.81)**
12 years	2.32 (2.23–2.40)	0.96 (0.91–1.00)	1.81 (1.68–1.94)	0.98 (0.90–1.06)
More than 12 years	1	1	1	1
*Social and community context*				
Unmarried	4.35 (4.21–4.50)	**2.31 (2.21–2.41)**	2.49 (2.34–2.65)	**1.85 (1.70–2.02)**
Parity				
Nulliparity	1	1	1	1
1 prior live birth	0.88 (0.85–0.92)	**1.17 (1.11–1.24)**	1.45 (1.32–1.60)	**1.88 (1.66–2.12)**
2 prior live births	1.59 (1.52–1.66)	**1.82 (1.69–1.96)**	4.00 (3.61–4.35)	**4.05 (3.54–4.63)**
3 or more prior live births	2.25 (2.14–2.36)	**2.31 (2.11–2.52)**	6.80 (6.20–7.45)	**5.40 (4.64–6.27)**
*Economic stability*				
Income category (per year)				
< $20,000	5.01 (4.79–5.23)	**1.70 (1.61–1.81)**	3.13 (2.87–3.41)	**1.47 (1.30–1.66)**
$20,000–40,000	3.09 (2.95–3.23)	**1.54 (1.46–1.62)**	2.47 (2.25–2.72)	**1.44 (1.29–1.61)**
$40,000–60,000	1.83 (1.73–1.93)	**1.32 (1.25–1.40)**	1.72 (1.53–1.93)	**1.30 (1.14–1.47)**
$60,000–85,000	1.21 (1.15–1.29)	**1.16 (1.09–1.23)**	1.31 (1.15–1.48)	**1.23 (1.08–1.41)**
> $85,000	1	1	1	1
Income dependents				
1 (self)	3.15 (2.97–3.34)	**1.55 (1.45–1.66)**	2.00 (1.76–2.27)	**1.32 (1.15–1.51)**
2	1	1	1	1
3	0.99 (0.96–1.03)	1.01 (0.95–1.07)	1.30 (1.18–1.43)	**0.85 (0.75–0.95)**
4	1.68 (1.61–1.76)	**1.31 (1.22–1.40)**	2.97 (2.70–3.26)	**1.21 (1.07–1.37)**
≥ 5	2.17 (2.06–2.28)	**1.42 (1.31–1.54)**	4.45 (4.06–4.91)	**1.44 (1.25–1.65)**
*Healthcare access and quality*				
Insurance (pre‐pregnancy)				
Private	1	1	1	1
Medicaid	3.96 (3.81–4.11)	**1.18 (1.12–1.25)**	2.78 (2.58–2.99)	1.04 (0.94–1.16)
Healthcare exchange	1.74 (1.60–1.91)	1.03 (0.93–1.13)	1.52 (1.26–1.83)	0.95 (0.78–1.17)
Other	3.10 (2.94–3.26)	**1.32 (1.24–1.42)**	2.33 (2.10–2.57)	**1.22 (1.08–1.39)**
None				
Pre‐pregnancy BMI (kg/m^2^)	1.50 (1.37–1.63)	1.08 (0.98–1.19)	1.20 (1.00–1.45)	1.06 (0.87–1.29)
< 18.5	1	1	1	1
18.5–25	1.13 (1.09–1.18)	1.00 (0.95–1.04)	1.30 (1.20–1.40)	**1.09 (1.00–1.18)**
25–29	1.39 (1.34–1.45)	1.03 (0.99–1.08)	1.62 (1.51–1.75)	**1.13 (1.04–1.23)**
≥ 30	2.37 (2.27–2.47)	**1.51 (1.43–1.58)**	2.15 (2.01–2.31)	**1.58 (1.45–1.72)**
Smoking history	1.04 (0.95–1.13)	0.82 (0.73–0.91)	1.22 (1.03–1.44)	0.77 (0.65–0.95)
Pre‐gestational diabetes	1.31 (1.24–1.41)	1.07 (0.98–1.16)	1.77 (1.59–1.98)	**1.21 (1.06–1.38)**
Chronic hypertension	1.88 (1.80–1.95)	**1.39 (1.32–1.47)**	1.93 (1.79–2.08)	**1.58 (1.45–1.73)**

*Note*: Bolded text indicates significant associations.

Abbreviations: aOR, adjusted odds ratio; BMI, body mass index; CI, confidence interval; OR, odds ratio.

^a^ *N* = 172,565; weighted population 8,866,281 with live singleton births from 43 states and the District of Columbia, excluding Arizona, California, Idaho, Nevada, Ohio, South Carolina, and Texas.

^b^ Adjusted for all variables shown.

^c^ The “other” race and ethnicity category refers to individuals identified as American Indian, Hawaiian, Alaska native, or mixed race.

**FIGURE 1:**
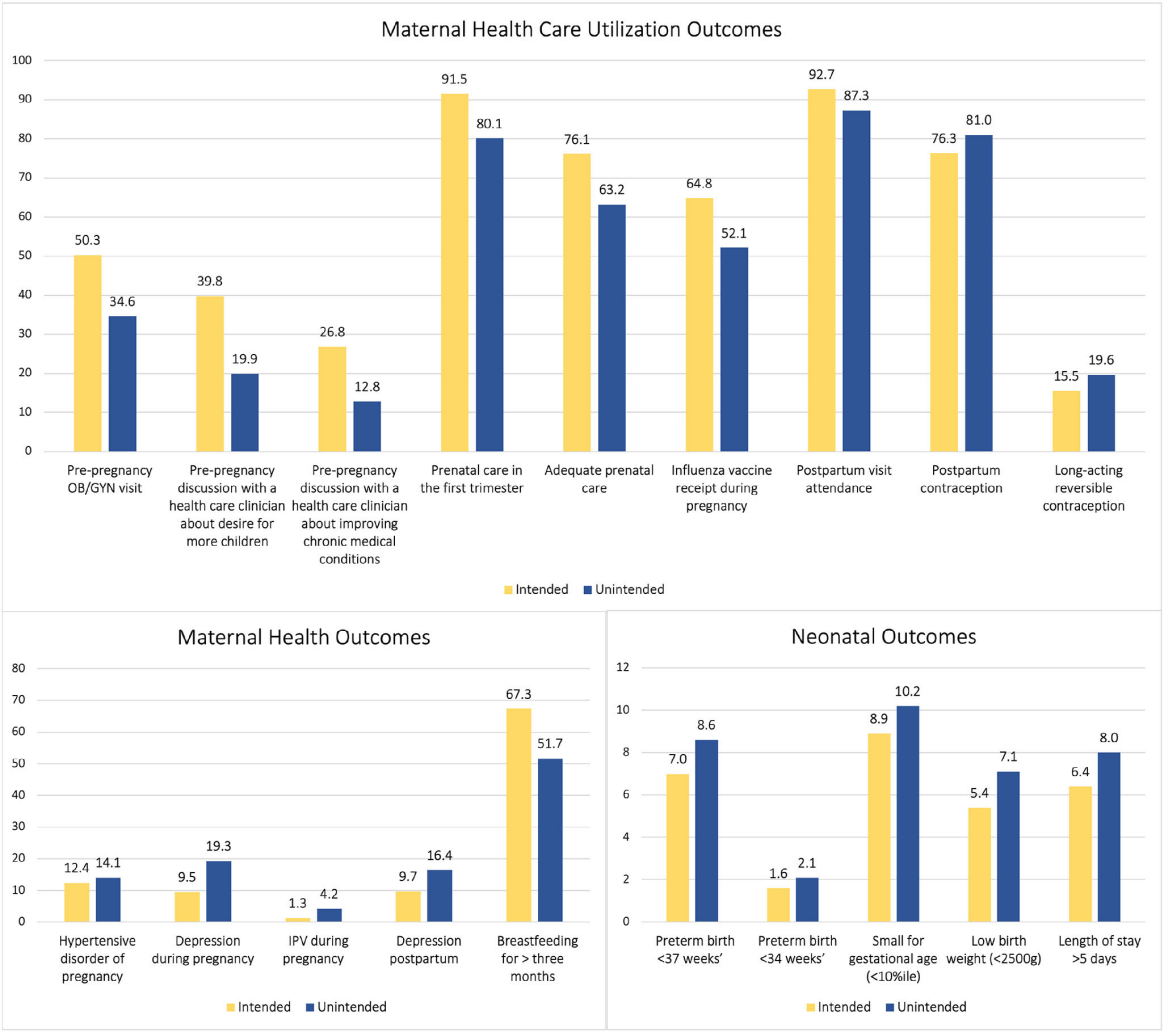
Healthcare utilization, maternal health, and neonatal outcomes by pregnancy intention group. IPV, intimate partner violence; OB/GYN, obstetrician gynecologist. Data presented as % *N* = 172,565; weighted population 8,866,281 with live singleton births from 43 states and the District of Columbia, excluding Arizona, California, Idaho, Nevada, Ohio, South Carolina, and Texas. All comparisons significant at *p* < 0.01. Pre‐pregnancy defined as any visit/discussion within 12 months prior to pregnancy. Adequate prenatal care as defined by the Kessner Adequacy of Prenatal Care Index.

For our second objective, unintended pregnancy was significantly associated with multiple adverse maternal and neonatal outcome metrics, including after accounting for potential confounders (Figure 1 and Table 3). With regards to healthcare utilization, compared to those with intended pregnancy, people with unintended pregnancy had significantly lower odds of engagement in pre‐pregnancy healthcare with an OB/GYN (aOR 0.79, 95% CI 0.76–0.82) or pre‐pregnancy discussions with a clinician about their desire for more children (aOR 0.57, 95% CI 0.55–0.59) or improving chronic health conditions (aOR 0.47, 95% CI 0.45–0.49). When pregnant, individuals with unintended pregnancy had lower odds of receiving prenatal care in the first trimester (aOR 0.60, 95% CI 0.57–0.63), receiving adequate prenatal care (aOR 0.75, 95% CI 0.72–0.78), receiving the influenza vaccine (aOR 0.86, 95% CI 0.83–0.89), and attending a postpartum visit (aOR 0.88, 95% CI 0.83–0.94). However, individuals with unintended pregnancy had *higher* odds of using postpartum contraception (aOR 1.44, 95% CI 1.38–1.50) including LARC (aOR 1.13, 95% CI 1.08–1.18). Individuals with unintended pregnancy had higher adjusted odds of adverse maternal outcomes (Table 3), including IPV during pregnancy (aOR 1.44, 95% CI 1.26–1.65), depression both during pregnancy (aOR 1.53, 95% CI 1.43–1.65) and postpartum (aOR 1.29, 95% CI 1.22–1.37), and lower odds of breastfeeding duration of greater than three months (aOR 0.90, 95% CI 0.86–0.94). Unintended pregnancy was not associated with neonatal outcomes on adjusted analyses (Table 3).

**TABLE 3:** Multivariable regression models for relationship of unintended pregnancy with healthcare utilization, maternal health, and neonatal outcomes.^a^, ^b^

Outcome variable	OR (95% CI)	aOR (95% CI)^c^
*Maternal healthcare utilization outcomes*		
Pre‐pregnancy^d^ OB/GYN visit	0.52 (0.51–0.54)	**0.79 (0.76–0.82)**
Pre‐pregnancy^d^ discussion with a healthcare clinician about desire for more children	0.38 (0.36–0.39)	**0.57 (0.55–0.59)**
Pre‐pregnancy^d^ discussion with a healthcare clinician about improving chronic medical conditions	0.40 (0.38–0.42)	**0.47 (0.45–0.49)**
Prenatal care in the first trimester	0.38 (0.37–0.40)	**0.60 (0.57–0.63)**
Adequate prenatal care^e^	0.54 (0.52–0.56)	**0.75 (0.72–0.78)**
Influenza vaccine receipt during pregnancy	0.59 (0.57–0.61)	**0.86 (0.83–0.89)**
Postpartum visit attendance	0.54 (0.52–0.57)	**0.88 (0.83–0.94)**
Postpartum contraception	1.32 (1.28–1.38)	**1.44 (1.38–1.50)**
Long‐acting reversible contraception	1.34 (1.28–1.39)	**1.13 (1.08–1.18)**
*Maternal Outcomes*		
Hypertensive disorder of pregnancy	1.16 (1.11–1.21)	1.05 (0.99–1.11)
Depression during pregnancy	2.28 (2.19–2.39)	**1.53 (1.43–1.65)**
IPV during pregnancy	3.12 (2.82–3.46)	**1.44 (1.26–1.65)**
Depression postpartum	1.18 (1.74–1.90)	**1.29 (1.22–1.37)**
Breastfeeding for > 3 months	0.52 (0.50–0.54)	**0.90 (0.86–0.94)**
*Neonatal Outcomes*		
Preterm birth < 37 weeks’	1.24 (1.19–1.31)	1.01 (0.95–1.08)
Preterm birth < 34 weeks’	1.26 (1.16–1.36)	0.91 (0.83–1.01)
Small for gestational age (< 10%ile)	1.16 (1.11–1.22)	1.04 (0.97–1.10)
Low birth weight (< 2500 g)	1.33 (1.27–1.39)	0.97 (0.90–1.05)
Length of stay > 5 days	1.26 (1.20–1.33)	0.99 (0.92–1.05)

*Note*: Bolded text indicates significant associations.

Abbreviations: aOR, adjusted odds ratio; CI, confidence interval; OB/GYN, obstetrician gynecologist; OR, odds ratio.

^a^ *N* = 172,565; weighted population 8,866,281 with live singleton births from 43 states and the District of Columbia, excluding Arizona, California, Idaho, Nevada, Ohio, South Carolina, and Texas.

^b^ Referent group is pregnant individuals with intended pregnancy.

^c^ Adjusted for maternal age, race and ethnicity, education, marital status, number of prior live births, annual household income, number of income dependents, pre‐pregnancy insurance, maternal body mass index, smoking, pregestational diabetes, chronic hypertension, and history of depression.

^d^ Pre‐pregnancy defined as any visit/discussion within 12 months prior to pregnancy.

^e^ Adequate prenatal care as defined by the Kessner Adequacy of Prenatal Care Index.

Results of the secondary analysis evaluating the three unintended pregnancy subgroups (mistimed, unsure, undesired) is shown in the Supporting Information. Each of the three unintended pregnancy subgroups were individually associated with higher odds of adverse outcomes when compared to intended pregnancy (Figure S1, Tables S3 and S4). In general, the mistimed and unsure subgroups had weaker associations (though still significant) whereas the undesired subgroup had the strongest associations with suboptimal healthcare utilization and adverse maternal health outcomes. Specifically, undesired pregnancy had stronger associations with IPV (aOR 1.93, 95% CI 1.58–2.37), depression during pregnancy (aOR 2.24, 95% CI 1.98–2.54), and postpartum depression (aOR 1.61, 95% CI 1.46–1.78). Undesired pregnancy was also associated with an increased risk of hypertensive disorders of pregnancy (aOR 1.26, 95% CI 1.14–1.40, Table S4).

## 4 DISCUSSION

In our analysis, 40.0% of respondents with a recent live birth reported that their pregnancy was unintended. We highlight social determinants of unintended pregnancy, including young age, non‐Hispanic Black race, and having fewer socioeconomic resources and more dependents. Unintended pregnancy was associated with greater likelihood of suboptimal healthcare utilization and adverse maternal outcomes. However, in our analysis, unintended pregnancy was not associated with adverse neonatal outcomes. In the secondary analysis of the different unintended pregnancy subgroups, undesired pregnancy was even more strongly associated with several social determinants of health and adverse maternal outcomes including hypertensive disorders of pregnancy.

Previous literature has evaluated maternal and neonatal outcomes in unintended pregnancies that end in a live birth, including studies using PRAMS [3, 4, 5, 6]. A recent systematic review and meta‐analysis demonstrated an association between unintended pregnancy and adverse maternal outcomes, however many of the studies and data included in this review were older (often more than ten years old), narrow in their evaluation of pregnancy outcomes, and restricted in their ability to control for potential confounders [5]. Prior studies have also suggested an association between unintended pregnancy and adverse neonatal outcomes [5, 13], however a more recent analysis by Mark et al. failed to find a significant association with multiple neonatal outcomes [15]. We corroborate many of these findings in a contemporary cohort (through 2020) and demonstrate associations between unintended pregnancy and additional outcomes including engagement in pre‐pregnancy healthcare, adequacy of prenatal care, receipt of the flu vaccine, postpartum contraception, and hypertensive disorders of pregnancy.

The mechanisms underlying the relationship between unintended pregnancy and adverse outcomes are not definitively known. As we and others have shown, unintended pregnancy is associated with increased socioeconomic stressors, and these social determinants have been associated with adverse health outcomes [16, 17, 18]. However, when comprehensively controlling for these potential confounders, we demonstrated an *independent* association between unintended pregnancy and several adverse maternal outcomes, suggesting that socioeconomic instability and disadvantage do not fully account for these relationships. Chronic stress is thought to contribute to the cumulative allostatic load of an individual, resulting in a physiological heightening of the neuroendocrine axis which may increase the risk for adverse health outcomes [19]. Antenatal perceived stress has been associated with adverse obstetrical outcomes, and therefore the chronic stress associated with continuing an unintended, and particularly, an undesired pregnancy, may partially underlie these associations [20]. Prospective assessments of these relationships including evaluation of maternal stress levels using validated tools are important areas of future work.

These contemporary results evaluating unintended pregnancy are especially important to consider given the decision in *Dobbs v. Jackson Women's Health Organization (*June 2022), after which 22 states completely banned or severely restricted abortion [21]. Although the rate of abortions has *increased* nationally since the *Dobbs* decision, due to broader availability of telehealth for medication abortion and the expansion of private “abortion funds,” the increased economic and logistical barriers of abortion care in ban states will result in the continuation of unintended pregnancies, particularly among socially disadvantaged populations [22, 23]. Even prior to the loss of federal protection, states with abortion restrictions, which were significantly less hostile than those in place today, had higher rates of unintended pregnancies continuing to term [24].

Our data highlight the importance of efforts to promote reproductive justice, thereby decreasing unintended pregnancy and illustrate potential areas for intervention. In addition to promoting safe and equitable access to abortion services, another area for targeted improvement is access to pre‐pregnancy healthcare. Contraception offers an effective preventive health strategy and insurance coverage is mandated under the Patient Protection and Affordable Care Act [25]. Access can be expanded by advocating for allowance of pharmacists to prescribe contraception, telehealth options for obtaining prescriptions, over‐the‐counter oral contraception without a prescription, and funding for Title X clinics which provide free contraception [26]. Additionally, legislative threats to reproductive freedom may one day expand to target contraception, and providers can utilize this data to advocate for the importance of maintaining access to safe and effective family planning methods.

Health systems and public programs should also engage in community outreach and education, particularly regarding the prospective assessment of circumstances leading to unintended pregnancy, implications of unintended pregnancy, and the accessibility of Title X clinics. We also need to promote clinical strategies to improve outcomes when unintended pregnancies are continued. Such strategies may include universal assessment for IPV and social determinants of health, expansion of social services and resources, enrollment of pregnant and postpartum individuals in publicly funded assistance programs, and comprehensive coverage of mental healthcare during pregnancy [27, 28, 29].

Our results must be interpreted within the context of several limitations inherent to the PRAMS survey design. The survey is administered to individuals with a recent live birth which excludes individuals who did not continue the pregnancy due to spontaneous or induced abortion. As such our results underestimate the overall rate of unintended pregnancy and are only reflective of those who continue pregnancy. Additionally, as the survey is administered in the postpartum setting, the results are inherently subject to recall and social desirability biases. We are also limited in our ability to generalize these results to the states that do not report to PRAMS. Finally, as PRAMS is a one‐time survey, we cannot report on long‐term implications of unintended pregnancy on maternal health, child development, or family socioeconomic trajectory all of which are important areas of future study.

An additional point of consideration is our choice to combine individuals who were unsure about pregnancy and who had mistimed pregnancies with individuals who had undesired pregnancies under the umbrella term of “unintended” pregnancy. While these subgroups may seem inherently different based on these responses, we elected to maintain the pregnancy intention groupings that have been previously established in the literature rather than exclude a large portion of the population from the analysis. We performed secondary analyses evaluating the separate subgroups within this category in order to further explore these differences. These results (shown in the Supporting Information) highlight how individuals in the mistimed and unsure subgroups have outcomes more similar to the undesired subgroup and the unintended pregnancy group as a whole rather than to the intended pregnancy group. These findings support the inclusion of the mistimed and unsure subgroups into the unintended pregnancy group and suggest that our primary analyses would not have been significantly different if either or both of these groups were excluded.

## 5 CONCLUSION

In summary, social determinants of health, including socioeconomic factors that represent financial stability, degree of socioeconomic responsibility to existing children and other dependents, and social support, are highly associated with unintended pregnancy. Unintended pregnancy is associated with suboptimal obstetric healthcare utilization and greater risk of maternal morbidity in this contemporary cohort. Findings highlight the importance of public health and policy initiatives that support family planning and the health of all pregnant individuals.

## CONFLICT OF INTEREST STATEMENT

The authors declare no conflicts of interest.

## Supporting information



Supporting information

Supplemental Figure 1: Healthcare utilization, maternal health, and neonatal outcomes by pregnancy intention including the three unintended pregnancy subgroups
OB/GYN, Obstetrician Gynecologist; IPV, intimate partner violence
Data presented as % N = 172,565; weighted population 8,866,281 with live singleton births from 43 states and the District of Columbia, excluding Arizona, California, Idaho, Nevada, Ohio, South Carolina, and Texas
All comparisons significant at P < 0.01
Pre‐pregnancy defined as any visit/discussion within 12 months prior to pregnancy
Adequate prenatal care as defined by the Kessner Adequacy of Prenatal Care Index

## Data Availability

The data that support the findings of this study are openly available in Pregnancy Risk Assessment Monitoring System at https://www.cdc.gov/prams/php/data‐research/index.html.
