# Supplementary material for: Risk factors for and outcomes after unintended pregnancy: Evaluation in a contemporary cohort
Source: Pregnancy (Hoboken). 2025 Jul 11;1(4):e70075. doi: 10.1002/pmf2.70075 (PMC13344767; doi:10.1002/pmf2.70075)
Supplement: Supplementary file 1 [file PMF2-1-e70075-s002.pdf]

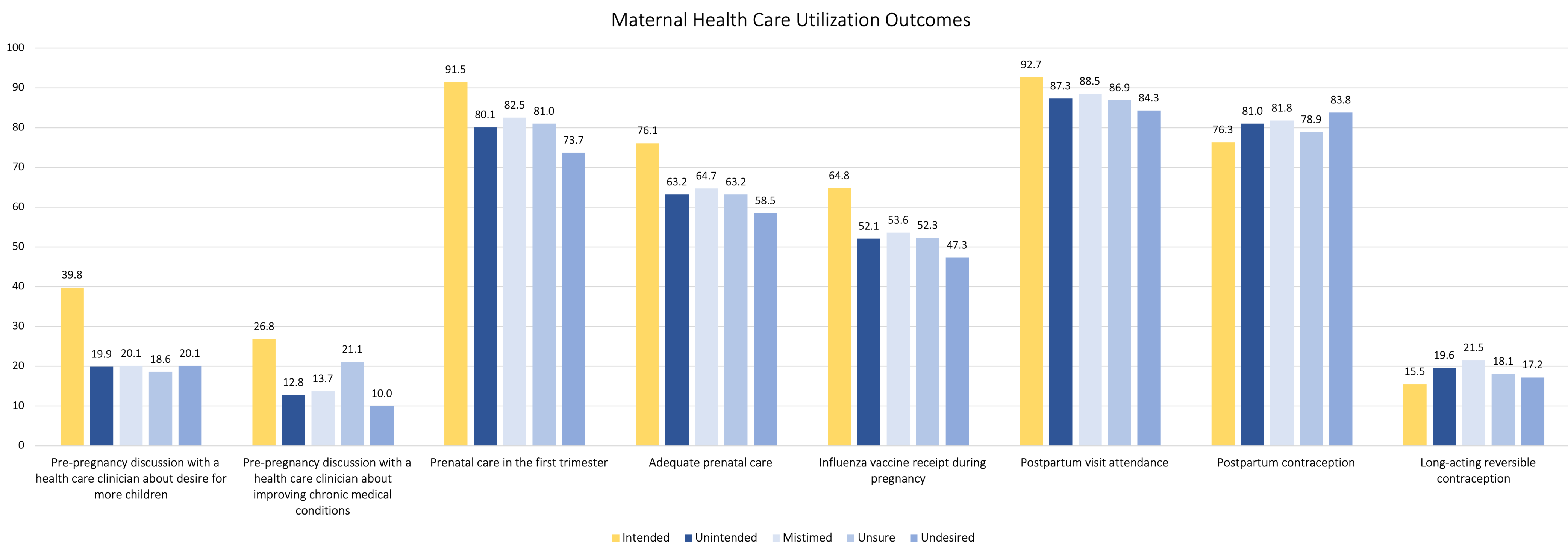

Maternal Health Care Utilization Outcomes
100
90
80
70
60
50
40
30
20
10
0
39.8
19.9
20.1
18.6
20.1
26.8
12.8
13.7
21.1
10.0
91.5
80.1
82.5
81.0
73.7
76.1
63.2
64.7
63.2
58.5
64.8
52.1
53.6
52.3
47.3
92.7
87.3
88.5
86.9
84.3
76.3
81.0
81.8
78.9
83.8
15.5
19.6
21.5
18.1
17.2
Pre-pregnancy discussion with a health care clinician about desire for more children
Pre-pregnancy discussion with a health care clinician about improving chronic medical conditions
Prenatal care in the first trimester
Adequate prenatal care
Influenza vaccine receipt during pregnancy
Postpartum visit attendance
Postpartum contraception
Long-acting reversible contraception
Intended
Unintended
Mistimed
Unsure
Undesired

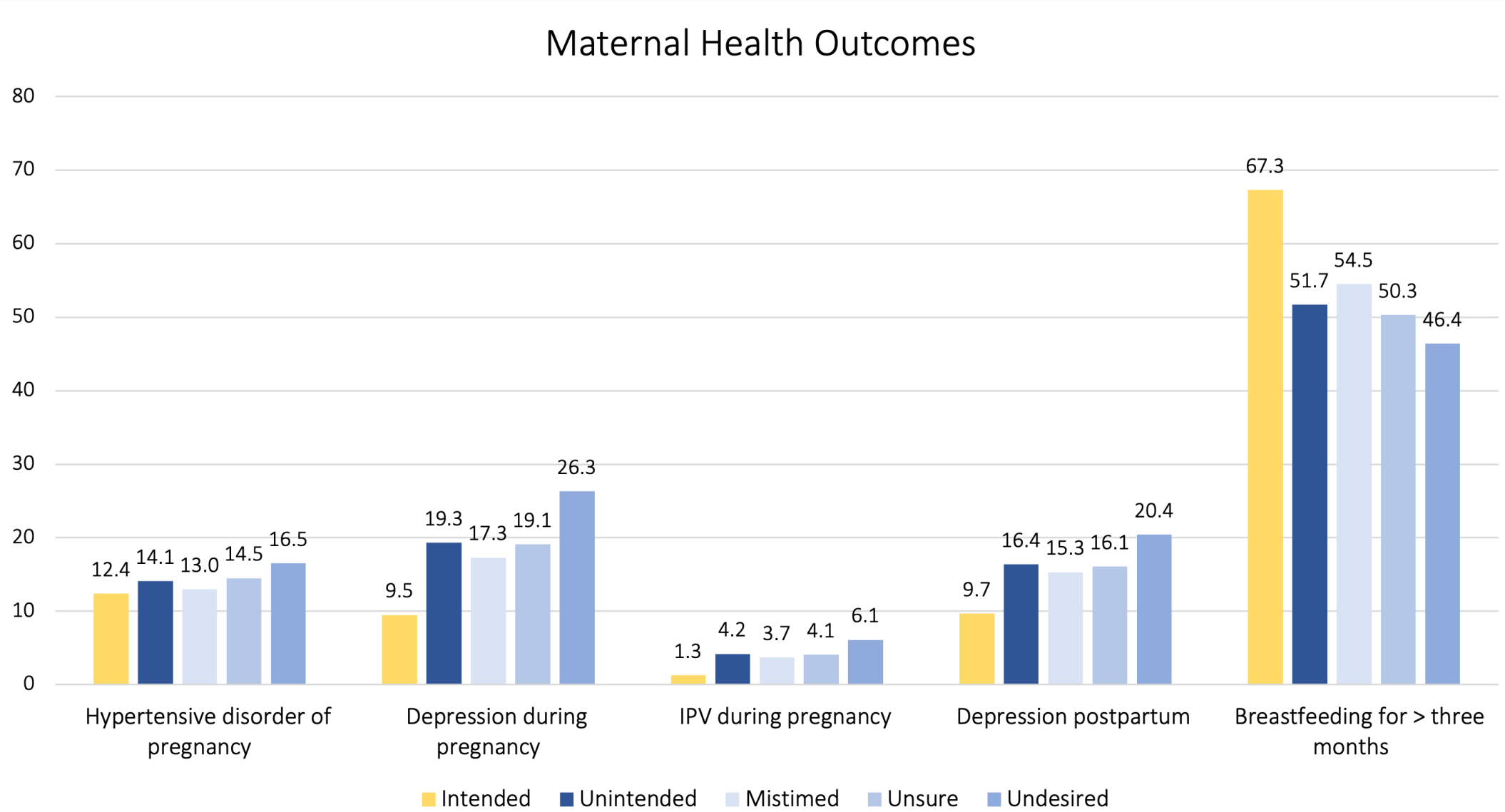

Maternal Health Outcomes
80
70
60
50
40
30
20
10
0
12.4
14.1
13.0
14.5
16.5
9.5
19.3
17.3
19.1
26.3
1.3
4.2
3.7
4.1
6.1
9.7
16.4
15.3
16.1
20.4
67.3
51.7
54.5
50.3
46.4
Hypertensive disorder of pregnancy
Depression during pregnancy
IPV during pregnancy
Depression postpartum
Breastfeeding for > three months
Intended
Unintended
Mistimed
Unsure
Undesired

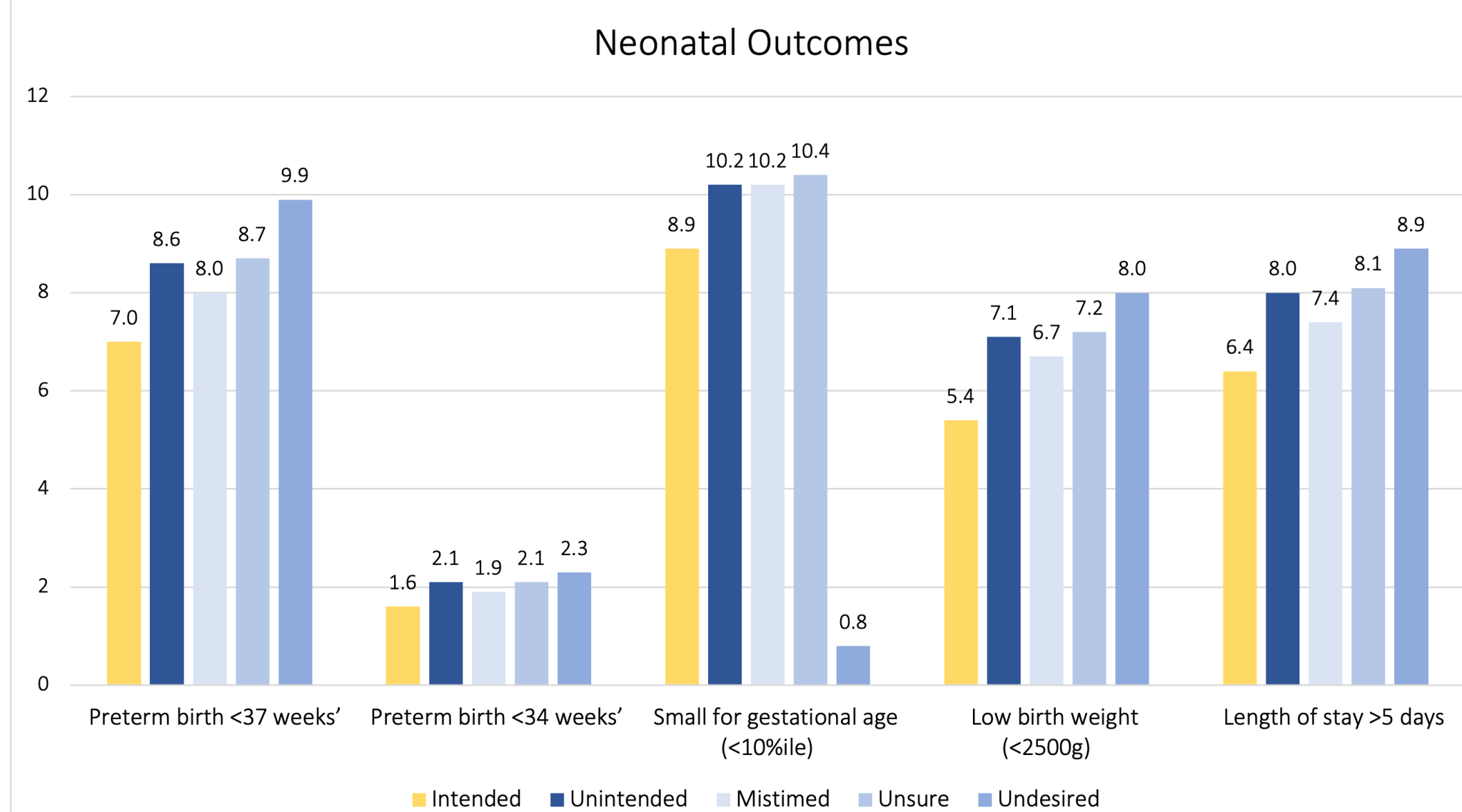

Neonatal Outcomes
12
10
8
6
4
2
0
7.0
8.6
8.0
8.7
9.9
1.6
2.1
1.9
2.1
2.3
8.9
10.2
10.2
10.4
0.8
5.4
7.1
6.7
7.2
8.0
6.4
8.0
7.4
8.1
8.9
Preterm birth <37 weeks’
Preterm birth <34 weeks’
Small for gestational age (<10%ile)
Low birth weight (<2500g)
Length of stay >5 days
Intended
Unintended
Mistimed
Unsure
Undesired
