# Supplementary material for: Risk factors for and outcomes after unintended pregnancy: Evaluation in a contemporary cohort
Source: Pregnancy (Hoboken). 2025 Jul 11;1(4):e70075. doi: 10.1002/pmf2.70075 (PMC13344767; doi:10.1002/pmf2.70075)
Supplement: Supplementary file 2 [file PMF2-1-e70075-s001.docx]

| Supplemental Table 1: States that met the response rate threshold by year of study period^*,†^ | | | | | |
| --- | --- | --- | --- | --- | --- |
| Site | 2016^‡,§^ | 2017^§^ | 2018^\|\|^ | 2019^\|\|^ | 2020^\|\|^ |
| Alabama | Χ | **√** | **√** | **√** | **√** |
| Alaska | **√** | **√** | **√** | **√** | **√** |
| Arizona | NR | Χ | Χ | Χ | **√** |
| Arkansas | **√** | Χ | **√** | **√** | **√** |
| Colorado | **√** | **√** | **√** | **√** | **√** |
| Connecticut | **√** | **√** | **√** | **√** | **√** |
| Delaware | **√** | **√** | **√** | **√** | **√** |
| District of Columbia | NR | Χ | **√** | **√** | **√** |
| Florida | Χ | Χ | Χ | **√** | **√** |
| Georgia | Χ | **√** | **√** | **√** | **√** |
| Hawaii | **√** | Χ | Χ | **√** | **√** |
| Illinois | **√** | **√** | **√** | **√** | **√** |
| Indiana | NR | Χ | **√** | Χ | Χ |
| Iowa | **√** | **√** | **√** | **√** | **√** |
| Kansas | NR | **√** | **√** | **√** | **√** |
| Kentucky | NR | **√** | **√** | **√** | **√** |
| Louisiana | **√** | **√** | **√** | **√** | **√** |
| Maine | **√** | **√** | **√** | **√** | **√** |
| Maryland | **√** | **√** | **√** | **√** | **√** |
| Massachusetts | **√** | **√** | **√** | **√** | **√** |
| Michigan | **√** | **√** | **√** | **√** | **√** |
| Minnesota | Χ | Χ | **√** | **√** | **√** |
| Mississippi | Χ | Χ | **√** | **√** | **√** |
| Missouri | **√** | **√** | **√** | **√** | **√** |
| Montana | NR | **√** | **√** | **√** | **√** |
| Nebraska | **√** | Χ | **√** | **√** | **√** |
| Nevada | NR | Χ | Χ | Χ | Χ |
| New Hampshire | **√** | **√** | **√** | **√** | **√** |
| New Jersey | **√** | **√** | **√** | **√** | **√** |
| New Mexico | **√** | **√** | **√** | **√** | **√** |
| New York | **√** | **√** | **√** | **√** | **√** |
| North Carolina | Χ | **√** | **√** | **√** | Χ |
| North Dakota | NR | **√** | **√** | **√** | **√** |
| Oklahoma | **√** | **√** | **√** | Χ | Χ |
| Oregon | Χ | Χ | **√** | **√** | **√** |
| Pennsylvania | **√** | **√** | **√** | **√** | **√** |
| Puerto Rico | NR | **√** | **√** | **√** | **√** |
| Rhode Island | **√** | **√** | **√** | **√** | Χ |
| South Dakota | Χ | **√** | **√** | **√** | **√** |
| Tennessee | Χ | Χ | Χ | **√** | **√** |
| Texas | Χ | Χ | Χ | Χ | Χ |
| Utah | **√** | **√** | **√** | **√** | **√** |
| Vermont | **√** | **√** | **√** | **√** | **√** |
| Virginia | **√** | **√** | **√** | **√** | **√** |
| Washington | **√** | **√** | **√** | **√** | **√** |
| West Virginia | **√** | **√** | **√** | Χ | **√** |
| Wisconsin | **√** | **√** | **√** | **√** | **√** |
| Wyoming | **√** | **√** | **√** | **√** | **√** |
| ^*^Green “**√**” indicates states with data available; Red “Χ” indicates states without data available; “NR” indicates states that were not reporting that year  ^†^California, Idaho, and Ohio were excluded as they did not report to PRAMS during the entire study period; South Carolina was excluded as their data is only available by direct request from the site and cannot be merged with the PRAMS data file  ^‡^In the year 2016 an additional 9 states and territories did not report to PRAMS: Arizona, Indiana, Kansas, Kentucky, Montana, Nevada, North Dakota, Washington, DC, and Puerto Rico  ^§^Minimum response rate for data release for 2016-2017 was 55%  ^\|\|^Minimum response rate for data release for 2018-2020 was 50% | | | | | |

| Supplemental Table 2: Social determinants of pregnancy intention in individuals with a live birth including unintended pregnancy subgroups^*†^ | | | | | |
| --- | --- | --- | --- | --- | --- |
|  | | | Unintended Subgroups | | |
| Social Determinant Categories and Factors | Intended  (60.0%) | Unintended  (40.0%) | Mistimed  (18.7%) | Unsure  (15.1%) | Undesired  (6.2%) |
| *Maternal Sociodemographic* |  |  |  |  |  |
| Age (years) |  |  |  |  |  |
| <20 | 1.5 | 7.2 | 8.8 | 3.8 | 4.1 |
| 20-30 | 42.3 | 54.3 | 61.8 | 49.6 | 43.1 |
| 30-40 | 52.5 | 35.7 | 28.7 | 40.2 | 46.0 |
| ≥40 | 3.6 | 2.8 | 0.8 | 3.8 | 6.8 |
| Race and ethnicity |  |  |  |  |  |
| Asian | 6.0 | 4.1 | 4.0 | 4.3 | 3.8 |
| Hispanic | 14.2 | 18.2 | 20.7 | 15.5 | 17.3 |
| Non-Hispanic Black | 9.2 | 22.7 | 21.1 | 22.7 | 27.7 |
| Non-Hispanic White | 67.1 | 50.0 | 50.0 | 52.3 | 46.5 |
| None of the above^‡^ | 3.5 | 4.7 | 4.3 | 5.2 | 4.8 |
| *Education Access and Quality* |  |  |  |  |  |
| Education |  |  |  |  |  |
| Less than 12 years | 7.2 | 13.2 | 12.2 | 14.5 | 13.3 |
| 12 years | 18.7 | 32.0 | 30.6 | 33.2 | 33.5 |
| More than 12 years | 74.1 | 54.8 | 57.2 | 52.4 | 53.2 |
| *Social and Community Context* |  |  |  |  |  |
| Unmarried | 23.4 | 57.1 | 57.0 | 56.9 | 57.8 |
| Parity |  |  |  |  |  |
| Nulliparity | 41.2 | 36.2 | 44.1 | 33.5 | 18.6 |
| 1 prior live birth | 36.8 | 28.5 | 31.0 | 27.8 | 22.8 |
| 2 prior live births | 14.0 | 19.5 | 15.4 | 21.1 | 28.1 |
| 3 or more prior live births | 8.0 | 15.8 | 9.4 | 17.6 | 30.6 |
| *Economic Stability* |  |  |  |  |  |
| Income category (per year) |  |  |  |  |  |
| <$20,000 | 14.6 | 35.5 | 35.0 | 35.5 | 36.8 |
| $20-40,000 | 16.4 | 24.5 | 24.3 | 24.3 | 25.3 |
| $40-60,000 | 13.2 | 11.7 | 12.0 | 11.3 | 12.0 |
| $60-85,000 | 13.1 | 7.7 | 7.8 | 7.5 | 7.7 |
| >$85,000 | 42.8 | 20.1 | 20.9 | 21.4 | 18.6 |
| Income dependents |  |  |  |  |  |
| 1 (self) | 5.7 | 13.6 | 15.3 | 13.2 | 9.6 |
| 2 | 37.4 | 28.3 | 31.8 | 33.7 | 18.9 |
| 3 | 33.8 | 25.4 | 27.6 | 24.0 | 21.9 |
| 4 | 14.1 | 17.9 | 15.1 | 18.8 | 24.2 |
| ≥5 | 9.1 | 14.9 | 10.3 | 16.2 | 25.4 |
| *Health Care Access and Quality* |  |  |  |  |  |
| Insurance (pre-pregnancy) |  |  |  |  |  |
| Private | 61.2 | 32.4 | 32.7 | 32.6 | 31.0 |
| Medicaid | 17.0 | 35.6 | 32.7 | 37.7 | 40.0 |
| Health care exchange | 3.7 | 3.4 | 3.5 | 3.5 | 3.4 |
| Other | 8.7 | 13.3 | 15.8 | 11.6 | 9.8 |
| None | 9.3 | 15.3 | 15.5 | 14.7 | 16.2 |
| Pre-pregnancy BMI (kg/m^2^) |  |  |  |  |  |
| <18.5 | 2.9 | 3.8 | 4.0 | 3.7 | 3.1 |
| 18.5-25 | 47.3 | 41.4 | 43.4 | 40.1 | 36.6 |
| 25-29 | 26.0 | 25.8 | 25.4 | 25.8 | 27.0 |
| ≥30 | 23.9 | 29.1 | 27.2 | 29.7 | 33.3 |
| Smoking history | 11.4 | 23.3 | 19.7 | 25.8 | 28.1 |
| Pre-gestational diabetes | 3.0 | 3.1 | 2.7 | 3.4 | 3.7 |
| Chronic hypertension | 4.6 | 5.9 | 4.8 | 6.3 | 8.4 |
| Depression history | 11.0 | 18.8 | 16.9 | 19.4 | 23.2 |
| BMI, body mass index  Data presented as %  ^*^N=172,565; weighted population 8,866,281 with live singleton births from 43 states and the District of Columbia, excluding Arizona, California, Idaho, Nevada, Ohio, South Carolina, and Texas  ^†^All comparisons significant at P<0.01, with the exception of pre-gestational diabetes  ^‡^The “none of the above” race and ethnicity category refers to individuals identified as American Indian, Hawaiian, Alaska native, or mixed race; these groups were collapsed due to insufficient sample size | | | | | |

| Supplemental Table 3. Multivariable regression models for relationship of unintended pregnancy subgroups with pre-pregnancy and pregnancy-related healthcare utilization^*†^ | | |
| --- | --- | --- |
| Outcome Variable | OR (95% CI) | aOR (95% CI)^‡^ |
| Pre-pregnancy^§^ OB/GYN visit |  |  |
| Mistimed | 0.56 (0.54-0.58) | **0.84 (0.80-0.89)** |
| Unsure | 0.49 (0.47-0.52) | **0.73 (0.70-0.77)** |
| Undesired | 0.50 (0.47-5.53) | **0.75 (0.70-0.81)** |
| Pre-pregnancy^§^ discussion with a health care clinician about desire for more children |  |  |
| Mistimed | 0.40 (0.38-0.42) | **0.60 (0.57-0.64)** |
| Unsure | 0.35 (0.33-0.36) | **0.52 (0.49-0.55)** |
| Undesired | 0.38 (0.35-0.41) | **0.58 (0.54-0.64)** |
| Pre-pregnancy^§^ discussion with a health care clinician about improving chronic medical conditions |  |  |
| Mistimed | 0.44 (0.41-0.46) | **0.52 (0.48-0.54)** |
| Unsure | 0.40 (0.37-0.42) | **0.46 (0.43-0.49)** |
| Undesired | 0.30 (0.27-0.33) | **0.35 (0.31-0.39)** |
| Prenatal care in the first trimester |  |  |
| Mistimed | 0.44 (0.41-0.46) | **0.65 (0.61-0.69)** |
| Unsure | 0.40 (0.37-0.42) | **0.62 (0.58-0.67)** |
| Undesired | 0.26 (0.24-0.28) | **0.45 (0.41-0.49)** |
| Adequate prenatal care^\|\|^ |  |  |
| Mistimed | 0.58 (0.55-0.60) | **0.77 (0.73-0.81)** |
| Unsure | 0.54 (0.51-0.56) | **0.75 (0.71-0.79)** |
| Undesired | 0.44 (0.41-0.47) | **0.67 (0.62-0.72)** |
| Influenza vaccine receipt during pregnancy |  |  |
| Mistimed | 0.63 (0.60-0.65) | **0.88 (0.84-0.92)** |
| Unsure | 0.60 (0.57-06.2) | **0.89 (0.83-0.91)** |
| Undesired | 0.49 (0.46-0.52) | **0.76 (0.71-0.82)** |
| Postpartum visit attendance |  |  |
| Mistimed | 0.61 (0.57-0.65) | **0.92 (0.85-0.99)** |
| Unsure | 0.53 (0.49-0.56) | **0.88 (0.82-0.95)** |
| Undesired | 0.43 (0.39-0.47) | **0.80 (0.72-0.88)** |
| Postpartum contraception |  |  |
| Mistimed | 1.40 (1.32-1.47) | **1.48 (1.40-1.56)** |
| Unsure | 1.16 (1.10-1.22) | **1.29 (1.22-1.36)** |
| Undesired | 1.60 (1.47-1.73) | **1.81 (1.66-1.98)** |
| Long-acting reversible contraception |  |  |
| Mistimed | 1.50 (1.43-1.58) | **1.21 (1.14-1.28)** |
| Unsure | 1.21 (1.14-1.28) | **1.08 (1.01-1.15)** |
| Undesired | 1.13 (1.05-1.24) | 0.96 (0.88-1.06) |
| OR, odds ratio; aOR, adjusted odds ratio; CI, confidence interval; OB/GYN, obstetrician and gynecologist  Bolded text indicates significant associations.  *N=172,565; weighted population 8,866,281 with live singleton births from 43 states and the District of Columbia, excluding Arizona, California, Idaho, Nevada, Ohio, South Carolina, and Texas  ^†^Referent group is pregnant individuals with intended pregnancy  ^‡^Adjusted for maternal age, race and ethnicity, education, marital status, number of prior live births, annual household income, number of income dependents, pre-pregnancy insurance, maternal body mass index, smoking, pregestational diabetes, chronic hypertension, and history of depression  ^§^Pre-pregnancy defined as any visit/discussion within 12 months prior to pregnancy  ^\|\|^Adequate prenatal care as defined by the Kessner Adequacy of Prenatal Care Index | | |

| Supplemental Table 4. Multivariable regression models for relationship of unintended pregnancy subgroups with maternal and neonatal health outcomes^*†^ | | |
| --- | --- | --- |
| Outcome Variable | OR (95% CI) | aOR (95% CI)^‡^ |
| *Maternal Outcomes* |  |  |
| Hypertensive disorder of pregnancy |  |  |
| Mistimed | 1.06 (1.00-1.12) | 0.97 (0.90-1.04) |
| Unsure | 1.20 (1.13-1.28) | **1.09 (1.01-1.17)** |
| Undesired | 1.40 (1.28-1.51) | **1.26 (1.14-1.40)** |
| Depression during pregnancy |  |  |
| Mistimed | 1.99 (1.88-2.10) | **1.45 (1.33-1.58)** |
| Unsure | 2.24 (2.12-2.34) | **1.40 (1.27-1.53)** |
| Undesired | 3.41 (3.16-3.68) | **2.24 (1.98-2.54)** |
| IPV during pregnancy |  |  |
| Mistimed | 2.72 (2.41-3.08) | **1.35 (1.15-1.57)** |
| Unsure | 3.02 (2.66-3.43) | **1.36 (1.15-1.61)** |
| Undesired | 4.62 (3.98-5.37) | **1.93 (1.58-2.37)** |
| Depression postpartum |  |  |
| Mistimed | 1.67 (1.58-1.77) | **1.21 (1.13-1.30)** |
| Unsure | 1.78 (1.68-1.90) | **1.27 (1.18-1.38)** |
| Undesired | 2.38 (2.19-2.58) | **1.61 (1.46-1.78)** |
| Breastfeeding for > three months |  |  |
| Mistimed | 0.58 (0.56-0.61) | 0.99 (0.94-1.04) |
| Unsure | 0.49 (0.47-0.51) | **0.74 (0.68-0.80)** |
| Undesired | 0.42 (0.39-0.45) | **0.86 (0.81-0.91)** |
| *Neonatal Outcomes* |  |  |
| Preterm birth <37 weeks’ gestation |  |  |
| Mistimed | 1.16 (1.09-1.23) | 1.00 (0.93-1.08) |
| Unsure | 1.26 (1.18-1.34) | 1.10 (0.98-1.22) |
| Undesired | 1.46 (1.33-1.60) | 0.99 (0.91-1.07) |
| Preterm birth <34 weeks’ gestation |  |  |
| Mistimed | 1.18 (1.07-1.30) | 0.95 (0.83-1.07) |
| Unsure | 1.28 (1.15-1.41) | 0.89 (0.78-1.02) |
| Undesired | 1.44 (1.24-1.66) | 0.89 (0.74-1.06) |
| Small for gestational age (<10%ile) |  |  |
| Mistimed | 1.16 (1.09-1.24) | 1.01 (0.93-1.09) |
| Unsure | 1.19 (1.11-1.27) | 1.05 (0.97-1.15) |
| Undesired | 1.11 (1.00-1.23) | 1.08 (0.96-1.22) |
| Low birth weight (<2500g) |  |  |
| Mistimed | 1.25 (1.18-1.32) | 0.96 (0.90-1.03) |
| Unsure | 1.36 (1.28-1.44) | 0.97 (0.91-1.05) |
| Undesired | 1.52 (1.40-1.65) | 1.05 (0.95-1.17) |
| Length of stay >5 days |  |  |
| Mistimed | 1.17 (1.09-1.24) | 0.88 (0.90-1.07) |
| Unsure | 1.30 (1.22-1.39) | 0.97 (0.89-1.06) |
| Undesired | 1.44 (1.31-1.58) | 1.03 (0.91-1.16) |
| OR, odds ratio; aOR, adjusted odds ratio; CI, confidence interval; IPV, intimate partner violence  Bolded text indicates significant associations.  *N=172,565; weighted population 8,866,281 with live singleton births from 43 states and the District of Columbia, excluding Arizona, California, Idaho, Nevada, Ohio, South Carolina, and Texas  ^†^Referent group is pregnant individuals with intended pregnancy  ^‡^Adjusted for maternal age, race and ethnicity, education, marital status, number of prior live births, annual household income, number of income dependents, pre-pregnancy insurance, maternal body mass index, smoking, pregestational diabetes, chronic hypertension, and history of depression | | |
